# Novel gas tungsten powder filler metal arc welding process

**DOI:** 10.1038/s41598-026-64307-z

**Published:** 2026-08-05

**Authors:** Badr El-Sayed, Eslam Syala, Ali El-Ashram, Jien-Wei Yeh, Abbas Anwar Ezzat, M. Essam El-Rafey

**Affiliations:** 1 Department of Materials Science, Institute of Graduate Studies and Researches (IGSR), Alexandria University, 163 Horreya Avenue, Shatby, 21526 Alexandria Egypt; 2 Department of Production Engineering, Faculty of Engineering, Alexandria University, Horreya Avenue, Shatby, 21544 Alexandria Egypt; 3 High Entropy Materials Center, Department of Materials Science and Engineering, National Tsing Hua University, Hsinchu, 30013 Taiwan

**Keywords:** Powder filler metal, GTPFAW, GTAW, Carbon steel S275 JR, Micro powder mixture, Welding, Engineering, Materials science

## Abstract

In the present investigation, an innovative and patented welding process referred to as gas tungsten powder filler metal arc welding (GTPFAW) has been developed, utilizing a micro powder mixture as a filler metal in lieu of conventional welding wires. This approach presents numerous advantages, encompassing a refined welding microstructure, enhanced mechanical properties, adaptability in composition tailored for various welding applications, and the elimination of the necessity for utilizing prefabricated welding wires. Steel grade S275 JR was employed as the foundational metal to be joined with the powder filler metal, which possessed a composition corresponding to that of the standard solid wire ER 70-S3. The evaluation of the weldability of gas tungsten powder filler metal arc welding (GTPFAW) methodology was conducted through a comprehensive analysis of the overall joint integrity and its corresponding mechanical attributes. The findings indicated that GTPFAW exhibited user-friendliness and yielded effective welding outcomes. The joint properties that resulted from the novel approach of GTPFAW were methodically contrasted with the joint properties generated by the traditional gas tungsten arc welding (GTAW) technique in combination with solid wire ER 70-S3, ensuring equivalent joint dimensions and the same steel parent metal classification S275 JR. The microstructural examination disclosed a significant acicular ferrite morphology characterized by its ultra-fine nature, markedly finer than the conventional microstructure produced by the solid filler. Furthermore, the weld metal produced exhibited a consistent hardness range (135–143 HV5) throughout its entire cross-section. This innovative technique possesses the capacity to enable a broad spectrum for the enhancement of filler metal formulations, thus providing substantial opportunities for the union of metals that display or possess particular or distinctive compositions. Moreover, this novel welding approach may be regarded as an environmentally sustainable practice and a proponent of the 2030 Agenda for Sustainable Development.

**Supplementary Information:**

The online version contains supplementary material available at 10.1038/s41598-026-64307-z.

## Introduction

A diverse array of sectors, including construction, manufacturing, and maritime industries, employ fusion welding technology. This welding process facilitates the formation of metal structures composed of either analogous or disparate metals into various geometrical configurations and dimensions. Typically, within the domain of fusion welding, the joint may exhibit either a homogeneous or heterogeneous composition throughout its entirety. Furthermore, in instances where a joining filler material with a distinct composition from that of the base metal is utilized, it is imperative that its melting point closely approximates that of the metals being joined^1^. A pivotal consideration for the welding engineer in ascertaining the most appropriate welding technique for a particular manufacturing environment is the ability of that technique to attain the necessary quality of the weld whilst concurrently reducing costs.

**Fig. 1:**
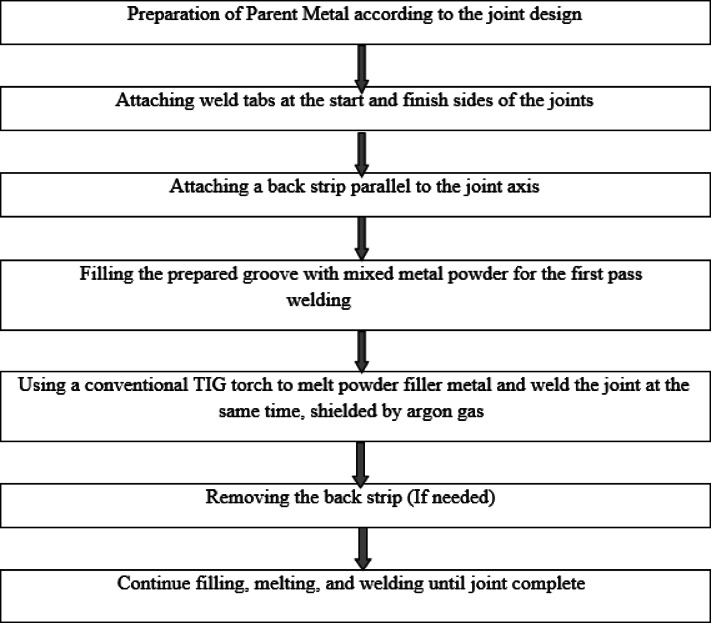
GTPFAW process sequence flowchart.

**Fig. 2:**
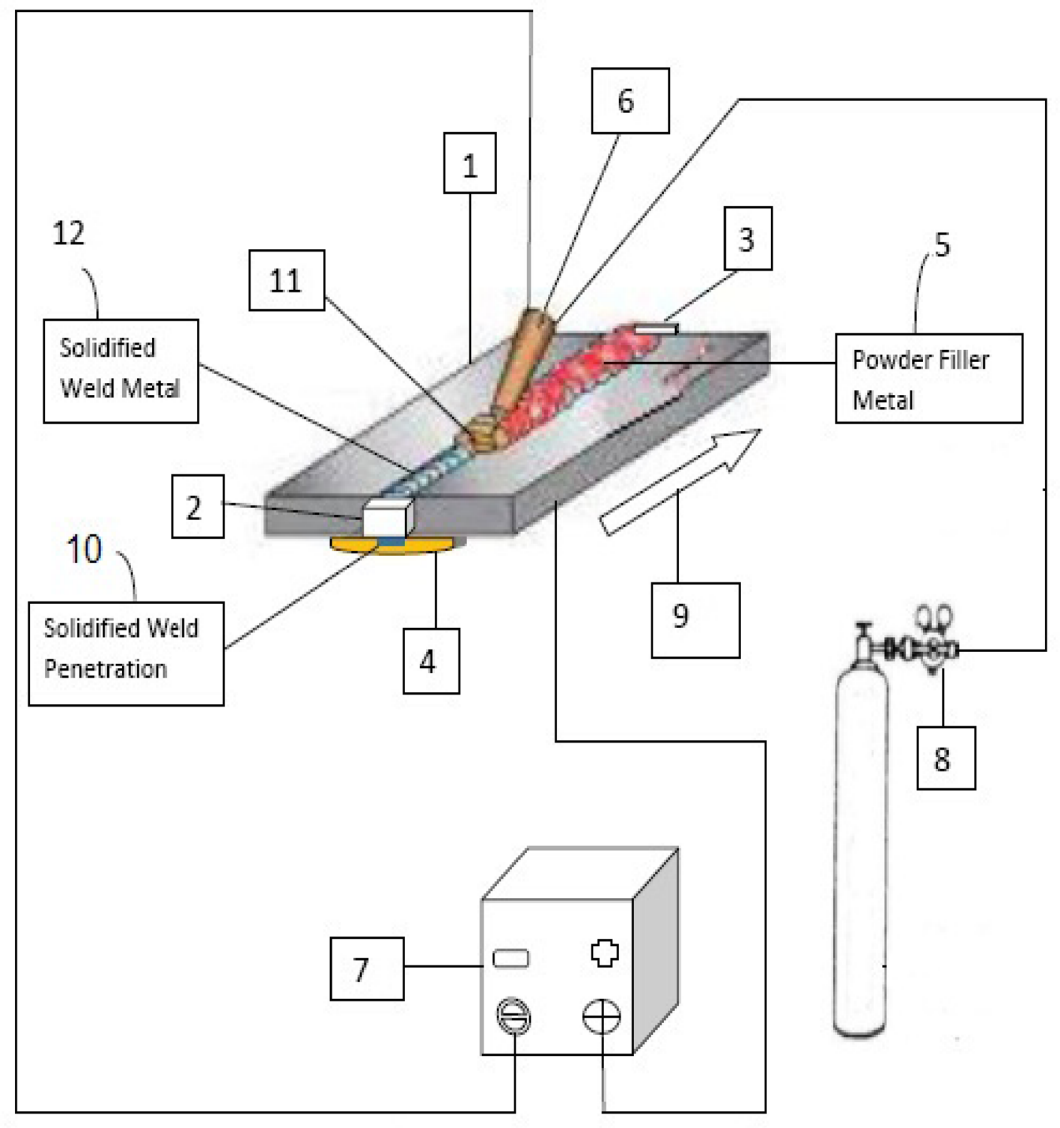
A schematic representation elucidating the elements and execution of the GTPFAW methodology. {1} parent metal, {2} prefabricated weld run tab, {3} weld off tab, {4} a back strip, {5} filled pre-mixed metal powder, {6} conventional TIG torch, {7} conventional power supply, {8} argon gas flow meter, {9} direction of welding progress, {10} root weld penetration, {11} electric arc melting and fuse the successive passes and {12} resultant weld metal.

**Fig. 3:**
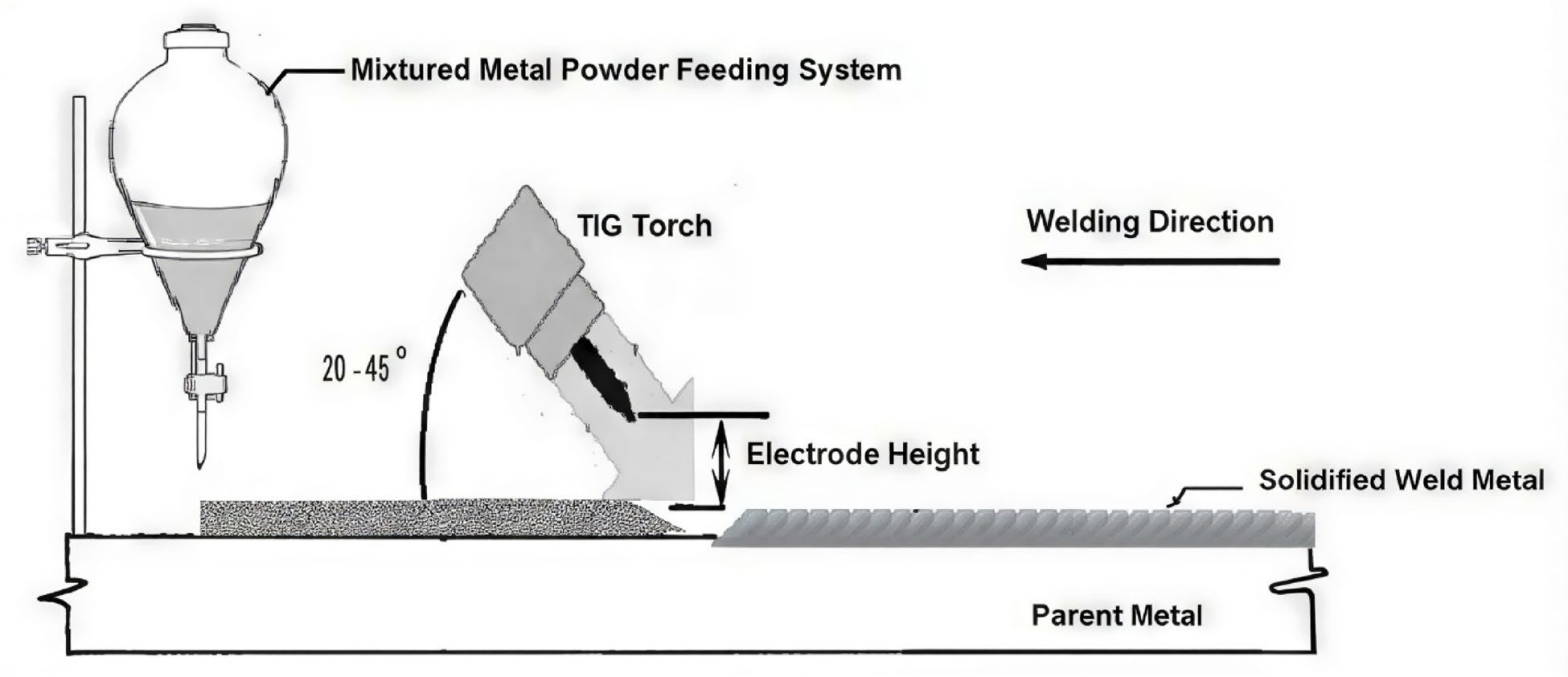
Torch manipulation during welding by GTPFAW process.

In the realm of conventional arc welding techniques, such as submerged arc welding (SAW), gas metal arc welding (GMAW), and gas tungsten arc welding (GTAW)/tungsten inert gas (TIG) methodologies, there exists a constrained spectrum of solid wire filler material compositions available. This limitation regarding the accessibility of solid filler metal compositions in the commercial sector is inherently associated with the restricted variety of filler metals that can be produced, which are subsequently defined in an array of national and international welding standards, alongside the specifications and technical documentation issued by manufacturers of solid wire^2–6^. The overarching parameters governing a fusion welding methodology encompass the energy derived from the thermal source, the quantifiable heat input values, and the efficacy of the weld pool shielding capability^1^. In the arc welding technique, an electric arc is initiated between either a consumable or non-consumable welding electrode and the base metals intended for connection, utilizing either a direct current (DC) or an alternating current (AC) power source. The elevated temperature at the interface of the electrode and the parent metal induces melting of the base metal, thereby facilitating the welding process and allowing for the advancement necessary to consummate the joint. Gas tungsten arc welding (GTAW), alternatively referred to as tungsten inert gas (TIG) welding, represents an arc welding process that employs a non-consumable tungsten electrode to establish a joint, either with or without the incorporation of filler material. Inert shielding gases such as argon or helium are utilized to protect the weld zone from atmospheric contaminants, while also ensuring the stability and continuity of the arc^7–11^. The presented (GTPFAW) process is a variation of the gas tungsten arc welding (GTAW) process in which conventional wire filler metal is substituted with a finely powdered material that is pre-placed within the joint gap. This technique operates as a manual arc welding technique, facilitating the fusion of metals through the thermal energy generated by an electric arc in conjunction with a powdered filler substance. The electric arc is precisely directed onto the base metal, traversing through the powdered filler and simultaneously melting both the filler and the base materials. The procedure does not necessitate any specialized power supply or torch, thereby rendering the setup relatively uncomplicated. It is crucial to underscore that this represents a modified technique, which is clearly differentiated from the conventional GTAW methodology. The welding sequence flowchart pertaining to the GTPFAW process is depicted in Fig. 1. Figure 2 provides a schematic representation of the GTPFAW methodology, illustrating the arcing cycle that transpires during the welding operation, in conjunction with the relevant consumable materials employed within this process. The parent metal {1} is prepared in accordance with the specified joint groove dimensions, subsequently tacked with prefabricated weld/run on tab {2} and weld/run off tab {3}. To facilitate the deposition of powder and achieve an optimal root penetration geometry, a back strip {4} is affixed beneath the joint and aligned parallel to the root gap. A filled pre-mixed metal powder is positioned within the groove to be welded, thus enabling the formation of the initial/root pass {5}. Manipulated through a conventional TIG torch {6} energized by a conventional GTAW/TIG electrical supply {7} and augmented with a flow of argon gas {8}. The torch is to be lowered toward the weld/run on tab until contact is made by the tungsten electrode with the tab. The torch is then to be promptly retracted to a short distance to establish the arc. The TIG torch, with the arc shielded by argon gas, is maneuvered toward the groove filled with powder, progressing in the direction of the weld/run off Table 9; the arc energy serves to melt the metal powder filler, thereby generating a molten weld pool that allows for fusion with the faying surfaces. Upon completion of the first/root pass, which ensures adequate weld penetration {10}, the back strip may either be removed or retained. To facilitate subsequent weld passes, the gap necessitates filling with the powder mixture and welding in a sequential manner until the surface is attained. For the construction of a reinforced cap of any specified height, additional powder can be introduced and fused in a similar manner {11}. Consequently, a joint of the type characterized by complete joint penetration weld can be successfully achieved {12}. In contrast, Fig. 3 delivers a representation of the manipulation of the torch and its angle of inclination in relation to the joint. A recurring challenge encountered by welding engineers employing the traditional Gas Tungsten Arc Welding (GTAW) technique pertains to the constrained availability, limited variety, and compatibility of commercially produced standard filler metals with respect to the specific base materials undergoing fusion and joining. To mitigate these limitations, the present investigation introduces an innovative methodology; Gas Tungsten Powder Filler Metal Arc Welding (GTPFAW) process, which enhances the design, selection, and preparation of suitable filler powders without being constrained by or dependent on the compositions of pre-manufactured welding wires. This innovation has the potential to transcend the current constraints associated with the development and evaluation of novel alloys. Moreover, the technique provides numerous advantages, such as enhanced adaptability in the selection of filler materials and more streamlined logistical processes. It is imperative to examine the attributes of the weld joints to ensure the productivity, durability, safety, and structural integrity of the welded components. It is of paramount importance to examine the localized change in properties of weld zones through the early identification of concealed defects, such as lack of fusion, cracks, or porosity, in order to avert catastrophic failures such as brittle fracture. Consequently, various non-destructive testing (NDT) methodologies were employed to ascertain the integrity and quality of the joints fabricated utilizing the novel welding technique. The rapid thermal cycles experienced during the welding process induce microstructural transformations that ultimately dictate the performance of the joint. The toughness and ductility of the material are profoundly influenced by phase transformations and grain growth, often referred to as granulation or refinement. Hence, metallurgical analyses were undertaken on the weld zone to examine the metallurgical properties of the joints produced via the innovative technique. Mechanical testing yields the empirical data necessary for the evaluation and assessment of the joints. To evaluate the load-bearing capacity of the GTPFAW joint and to identify localized regions of hardening or softening, tensile strength and hardness tests were performed. Moreover, an impact toughness test was executed to assess the joint’s susceptibility to brittle failure, particularly under conditions of dynamic loading. Furthermore, in order to conduct a comparative analysis of the metallurgical characteristics of the weld metal obtained through the innovative technique utilizing a powder exhibiting a chemical composition similar to that of solid wire ER 70-S3, in contrast to the properties resulting from the weld metal generated by the conventional methodology employing solid wire ER 70-S3, a sample weld was executed under identical dimensions and utilizing the same type of base metal, S275 JR.

## Experimental procedure

**Fig. 4:**
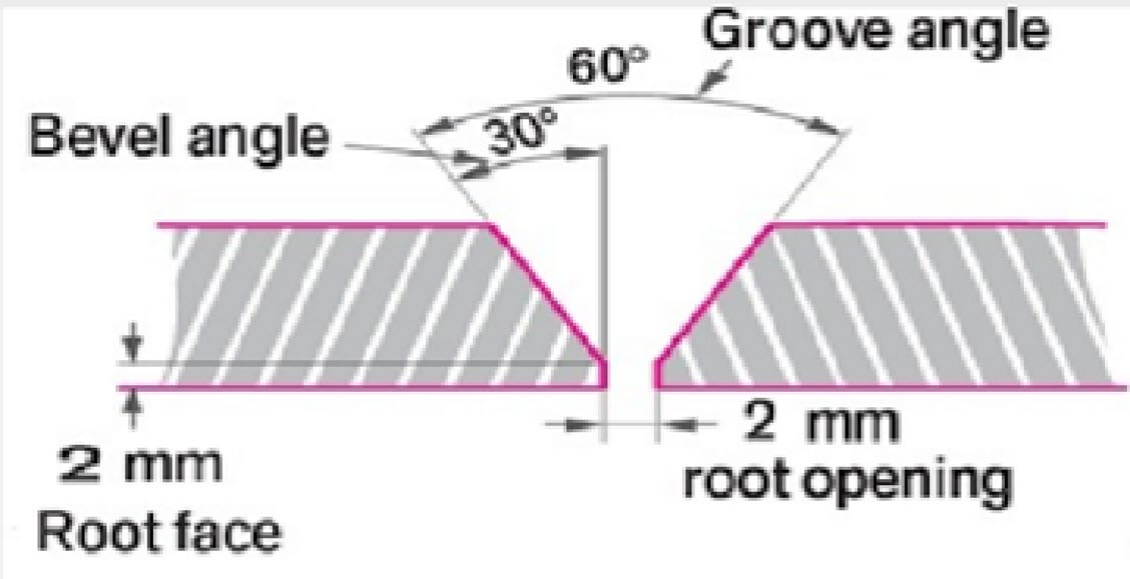
The dimensions of the two base metals joint to be welded.

The investigation of weldability in the present research was conducted on a steel plate of 5 mm thickness (base metal) exhibiting a strength of 459 N/mm², classified as steel grade S 275JR, in accordance with EN10025 standard^12^. The compositional percentages and mechanical properties of the plate are delineated in Table 1. Two base metal plates, measuring 600 mm and 400 mm in both length and width, respectively, were fabricated employing a motor equipped with cutting and grinding discs to mitigate the generation of excessive heat. The joint geometry that was prepared featured a groove angle of 60° (bevel angle of 30°), along with a root face and root gap of 2 mm, as depicted in Fig. 4.

**Table 1:** Chemical composition and tensile properties of plate S 275JR, 5 mm thickness.

Plate	Chemical Composition (wt%)									Mechanical properties	
5 mm	C	Si	Mn	P	S	Cr	Ni	Mo	V	Yield strength N/mm^2^	Tensile strength N/mm^2^
	0.15	0.02	0.47	0.008	0.023	0.09	0.1	0.017	0.004	312	459

**Table 2:** Weight percentages of the metal powder that are compatible with AWS A5.18 requirements for ER70S-3 solid wire^13^.

Reference	Weight%										
	C	Si	Mn	S	*P*	Cr	Ni	Mo	V	Cu ^(1)^	Fe
AWS A5.18 requirements for solid wire, ER70S-3	0.06–0.15	0.45 -0.75	0.9 -1.4	0.035 max.	0.025 max.	0.15 max	0.15 max	0.15 max	0.03 max	0.50 max	Remain
Prepared metal powder mixture	0.1	0.5	1.5	0.005	0.017	0.15	0.15	0.15	--	--	97.4

(1) Copper (Cu) was not added to the mixed powder, where it was used as a coat of solid wire for corrosion prevention purposes.

**Fig. 5:**
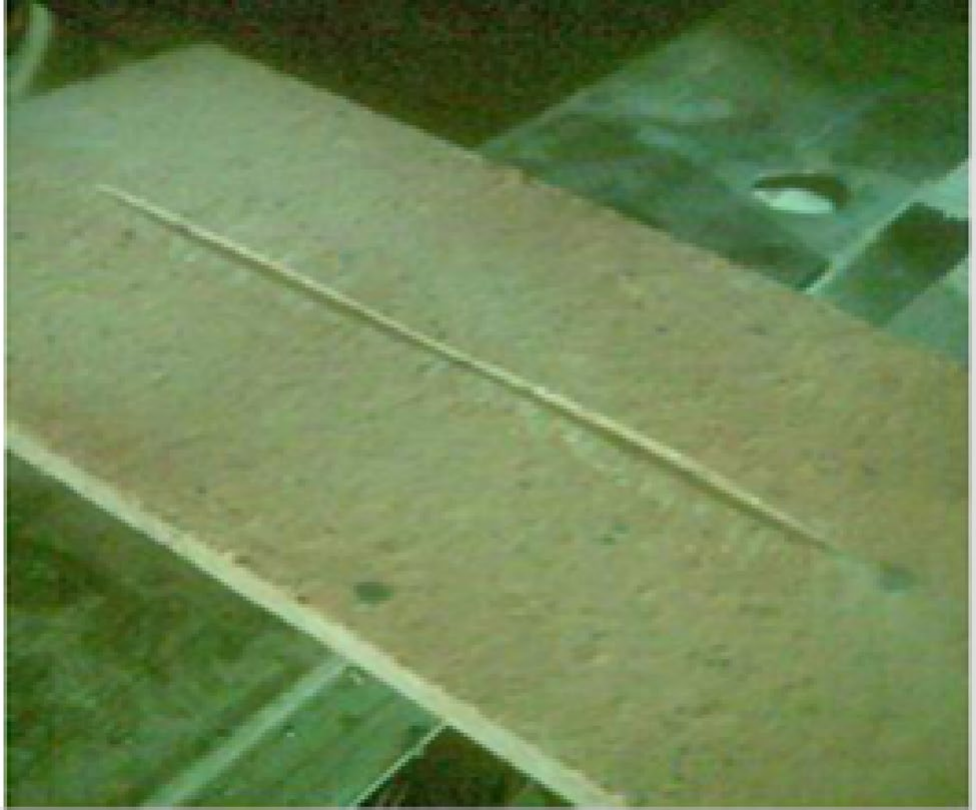
Notched thermal brick used as a back strip during welding for the GTPFAW joint.

Run-on/off tabs, which were constructed from the identical base metal plate, were affixed to the joints. Elemental metal powder was procured from the Egyptian Swedish Welding Electrodes Company (ESWECO) - HEDO Group. This powder is specifically engineered to serve as an alloying component for electrode coatings utilized in the shielded metal arc welding process (SMAW) consumables. The metal powders utilized in the experiment exhibit a granular morphology, with a mean particle size of approximately 100 μm, whereas the element carbon was utilized in its particulate state as carbon dust during the process of powder amalgamation/mixing. All elemental powders possess a purity exceeding 99%. The formulation of the prepared filler metal mixture conformed to the specified composition of ER70S-3 solid wire in accordance with AWS A5.18 classification standard, as illustrated in Table 2^13^. The mixing process was executed utilizing a mechanical mixer for a duration of 30 min at a rotational speed of 60 rpm. The interstice of the joint was occupied with the mixed powder, wherein a thermal brick measuring 20 mm in thickness, featuring a notched surface as depicted in Fig. 5, was employed as a backing strip beneath the entire length of the joint and aligned parallel to the root gap throughout the welding process to provide support for the composite powder and to achieve optimal root penetration geometry. A multi-pass weld was executed in a flat position (1G) utilizing a welding power supply characterized by an output range of 60–500 Amperes. The arc was expertly manipulated and controlled by an air-cooled welding torch that utilized a 2% Thoriated non-consumable tungsten electrode with a vertex angle of 30°. During the welding process, argon gas (Ar_(g)_) of 99.9% purity was utilized as a shielding gas, delivered through a ceramic gas nozzle with an inner diameter of 9.5 mm and regulated by a gas flow meter. No preheating, tack welds, Jigs, or fixtures were employed.

**Fig. 6:**
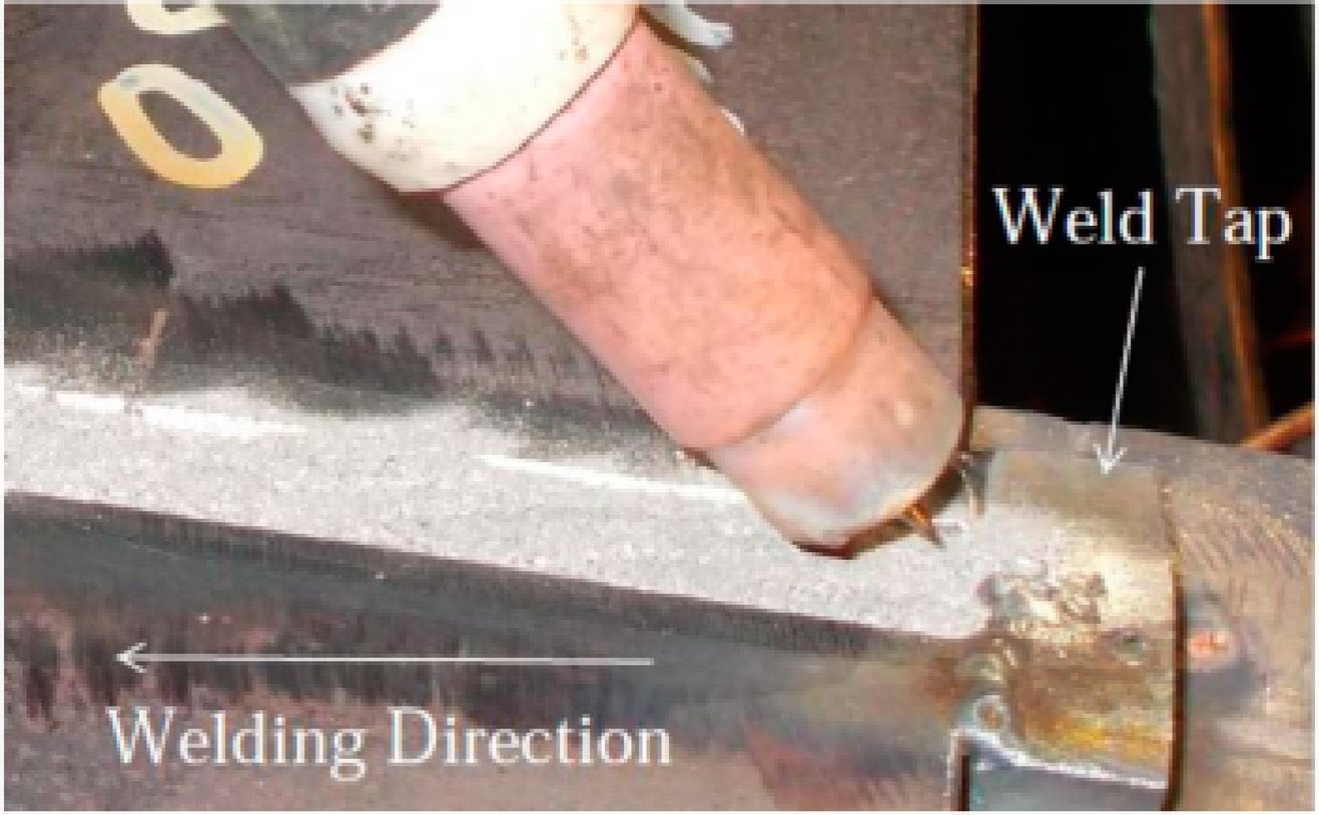
Welding of groove joint filled with metal powder mixture at pass no.4.

**Fig. 7:**
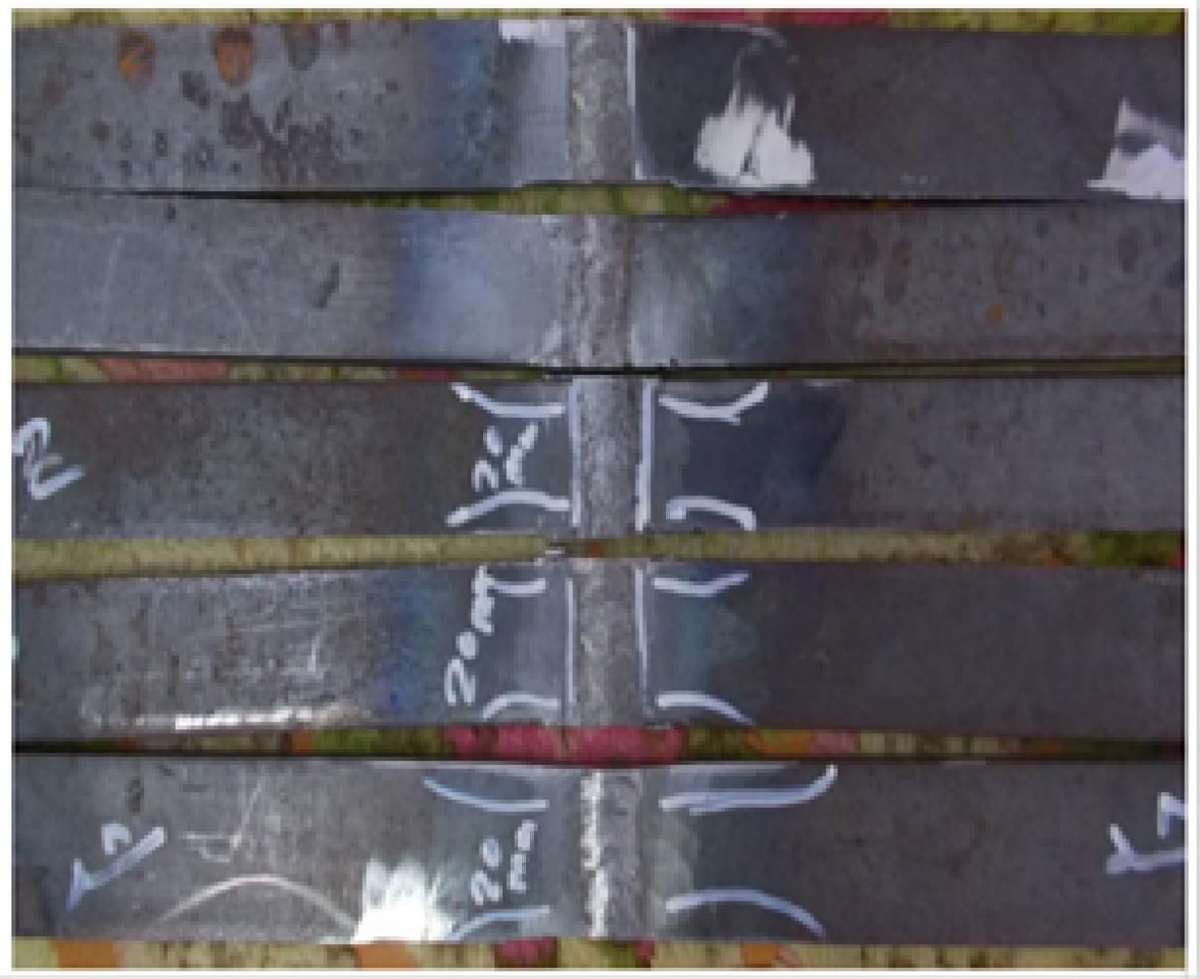
Specimens of GTPFAW Joint extracted for tensile test.

**Fig. 8:**
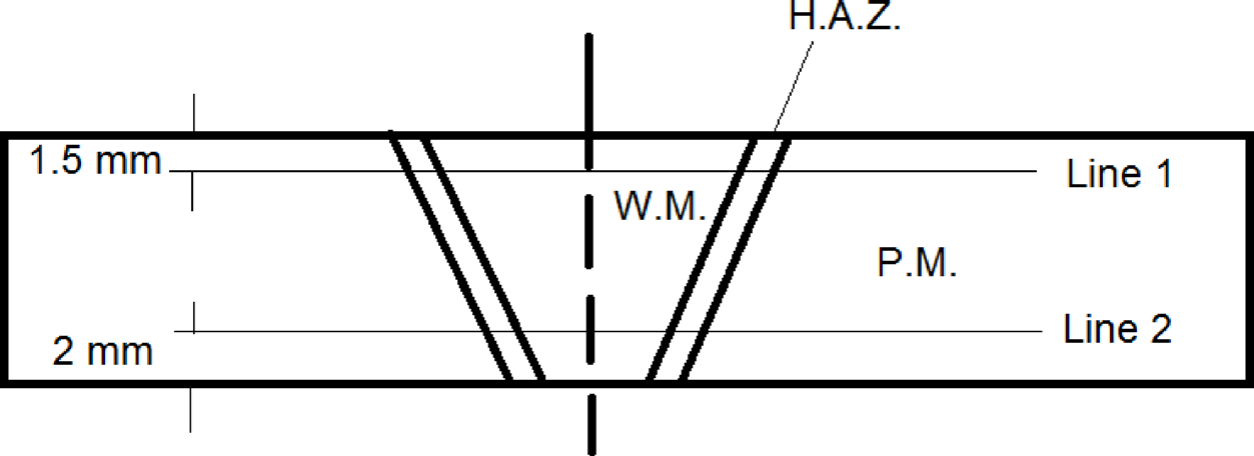
Locations of hardness measurement at weld metal (W.M.), heat-affected zone (H.A.Z.), and parent metal (P.M.) of GTPFAW joint.

Figure 6 represents an example of the powder welding methodology at the beginning of welding pass number four. The welding parameters documented during the welding of the joint using powder filler are illustrated in Table 3. The heat inputs during the welding process were computed in accordance with the recommendations set forth by the British Standard BS EN 1011-1, whereby the welding heat input was determined by multiplying the arc voltage and current by the thermal efficiency factor and subsequently dividing by the travel speed, with the process thermal efficiency considered to be 60%^14^. A standard solid wire, ER70S-3, known for its capability to weld S 275JR base metal^15^, was utilized for the joining of an analogous joint through the same apparatus, excluding the backing strip, to facilitate a comparative analysis of the resultant weld metal microstructure. The solid wire, possessing a diameter of 2.4 mm, exhibited a chemical composition as delineated in Table 4 based on its certification. The joint gap was completed with three passes, as the heat input was within the range of 0.58 to 1.15 kJ/mm. The welding parameters recorded for the joint welded with solid wire are presented in Table 5. The entirety of the GTPFAW joint length underwent non-destructive examination to assess the quality of both the surface and the internal structure of the weld. Various examination techniques, including visual inspection, color contrast dye penetrant, and radiography methods, were employed. Additionally, destructive tests were performed to ascertain the mechanical and metallurgical properties, with test specimens extracted from the completed joint in accordance with the AWS D1.1^16^ requirements. Five sub-sized tensile specimens were excised from the joint in a direction perpendicular to the weld axis, as shown in Fig. 7, and subsequently machined into a dog-bone configuration consistent with the AWS D1.1 specifications. The tensile testing was conducted using a universal tensile testing apparatus, Model EM2-600-FR, with a crosshead speed of 2 mm/min. Metallographic investigation was performed on five specimens, which were etched with 2% and 5% Nital for micrographic and macrographic examination, respectively. An optical reflected light microscope, Olympus BX 41 M-LED, was used for the observation of the microstructure of the resultant weld metal, which had been polished using 0.3 μm alumina. The Image J-1.46r software was utilized for the quantification of the grain size of the weld metal, with reference made to a micrograph of the weld metal magnified at 100X. A Zwick/Roell ZHU 250 hardness tester machine apparatus was utilized for the evaluation of the hardness of the resultant joint. An applied load of 5 kg was maintained for a duration of 15 s for each indentation. The microhardness assessment was executed on five specimens through the parent metal (P.M.), heat-affected zone (H.A.Z.), and weld metal (W.M.) of a macro-etched specimen, delineated by two lines, as illustrated in Fig. 8. Spectrometric chemical analyses of the solidified weld metal were performed on a cross-section of two coupons extracted from the GTPFAW welded joint, utilizing an ARL spectrometer, model 3460.

**Table 3:** Recorded welding parameters of the butt joint, welded by the new GTPFAW process, utilizing micro powder filler metal having a composition compatible with a solid ER 70 S-3 composition.

Pass	Polarity	Current (Ampere)		Voltage (Volt)		Travel speed (mm/s)	Heat Input (kJ/mm)		Argon gas flow rate (L/min)
		Min.	Max.	Min.	Max.		Min.	Max.	
1	DCEN	115	120	10	12	1.11	0.62	0.78	8
2	DCEN	110	140	11	13	1.05	0.69	1.04	7
3	DCEN	105	120	12	14	1.00	0.76	1.01	8
4	DCEN	140	160	10	12	1.11	1.01	1.38	6
5	DCEN	110	130	10	12	1.25	0.70	1.00	6

**Table 4:** Chemical composition of solid wire ER 70 S-3, 2.4 mm in wt%.

C	Si	Mn	*P*	S	Cr	Ni	Mo	V	Cu	Al	Zr	Ti
0.088	0.69	1.3	0.004	0.011	0.024	0.018	0.005	0.008	0.077	0.006	0.008	0.009

**Table 5:** Recorded welding parameters of butt joint, welded by solid wire filler metal, ER70S-3, 2.4 mm diameter.

Pass	Filler form	Filler type	Polarity	Current (Amperage)		Voltage (Volt)		Travel speed (mm/s)	Heat Input (KJ/mm)		Ar. gas flow (L/min)
				Min	Max	Min	Max		Min	max	
1	Solid, 2.4^Ø^	70 S-3	DCEN	190	200	19	20	2.08	1.04	1.15	9
2	Solid, 2.4^Ø^	70 S-3	DCEN	200	220	20	22	4.17	0.58	0.70	12
3	Solid, 2.4^Ø^	70 S-3	DCEN	210	235	17	18	3.33	0.64	0.76	11

## Results

**Fig. 9:**
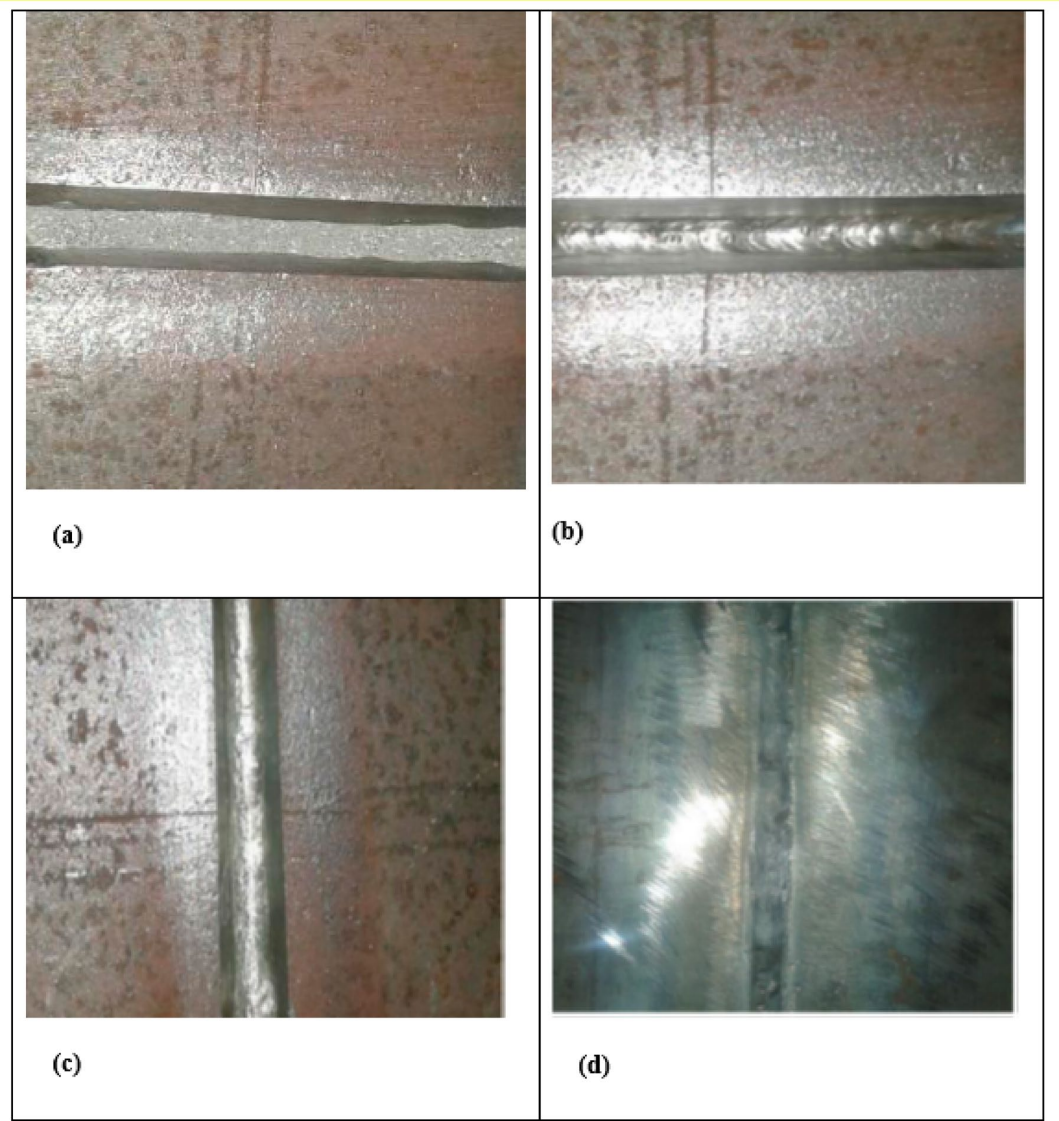
Obtained GTPFAW Joint; (**a**) an adequate powder filler metal distribution in the groove before welding; (**b**) acceptable fusion of powder filler metal; (**c**) the bonding of solidified filler metal and base metal; and (**d**) acceptable root penetration of the weld.

**Fig. 10:**
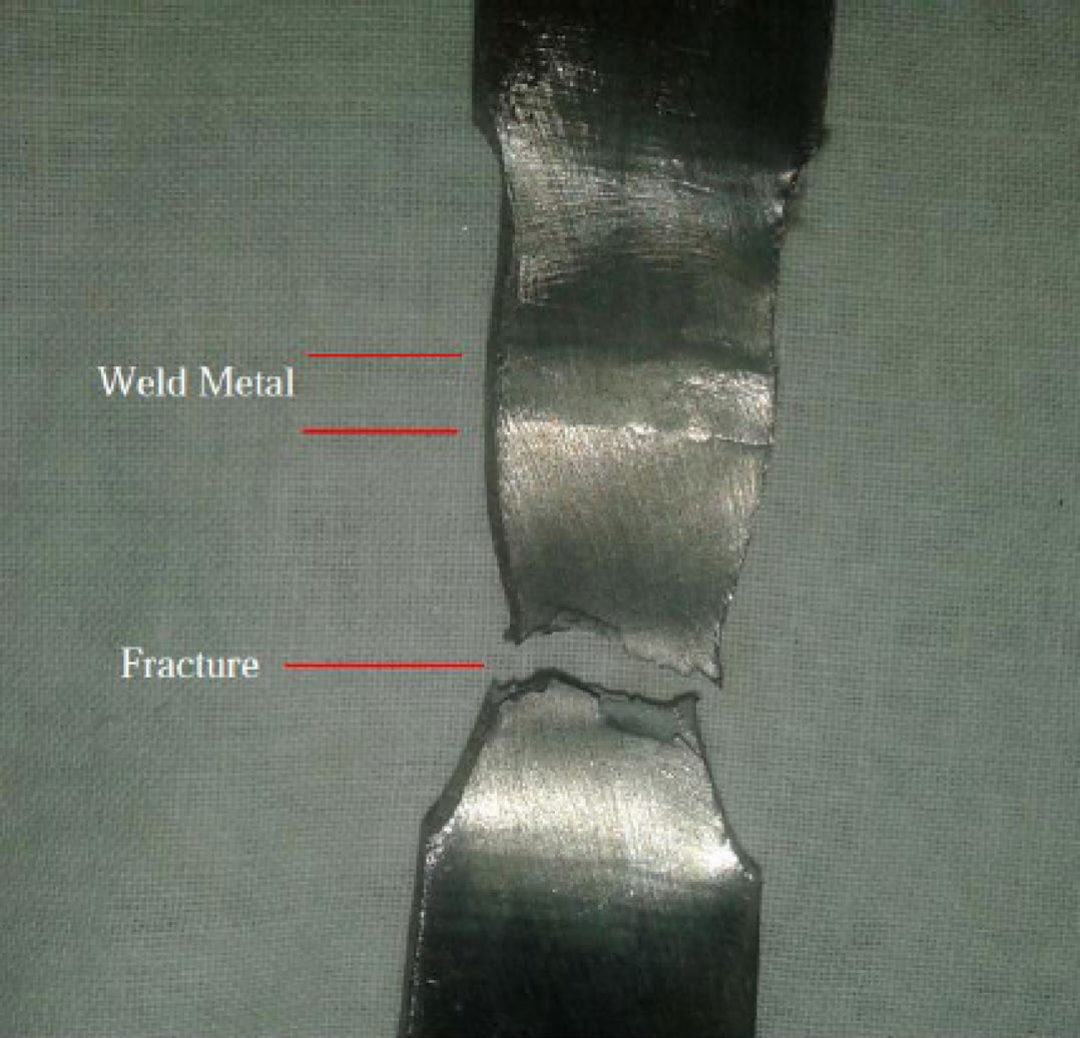
Fracture location in the base metal of a tensile specimen No.1 of the GTPFAW joint.

**Fig. 11:**
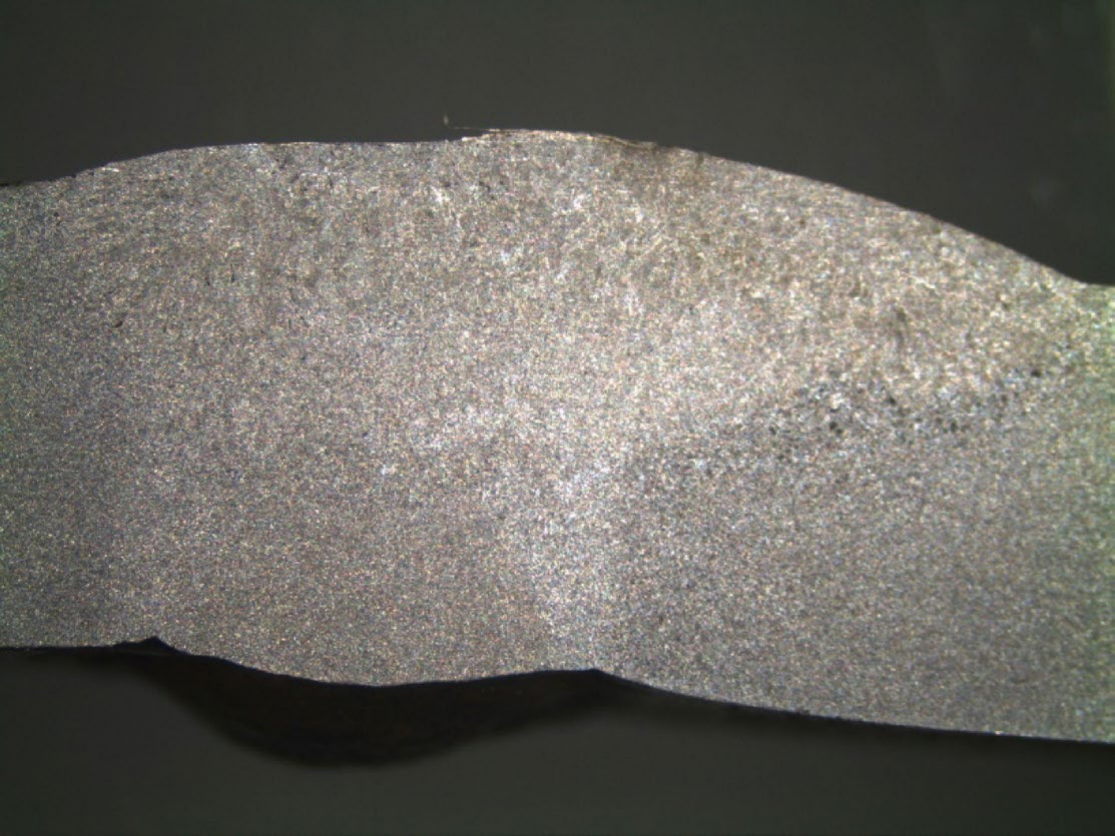
Macro metallography of complete joint cross-section at the widest cap of GTPFAW joint.

**Fig. 12:**
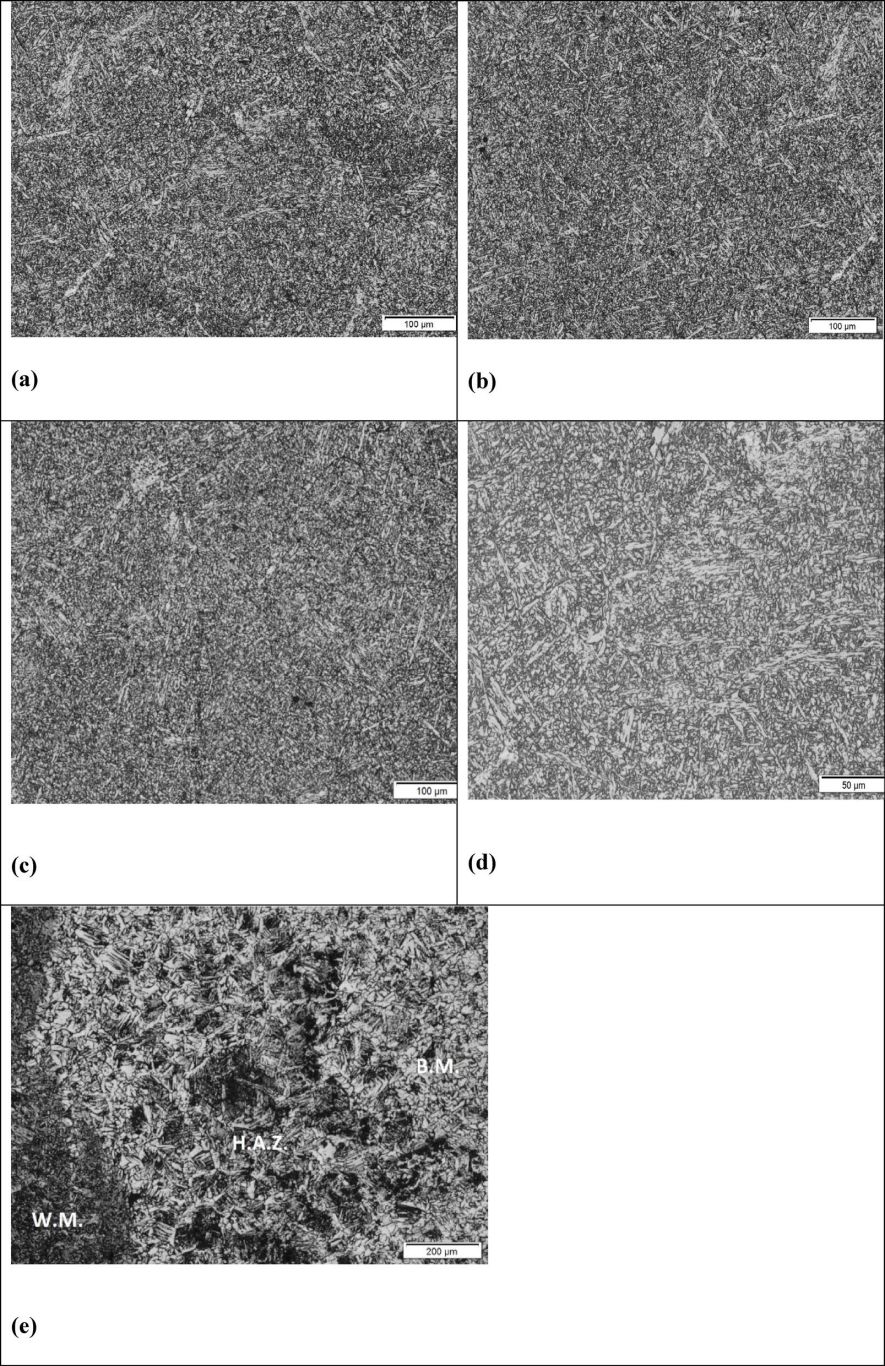
The microstructure of the resultant GTPFAW Joint. The microstructure of the weld metal at (**a**) the root and (**b**) cap zones, respectively; (**c**) and (**d**) the weld microstructure at the middle joint, and (**e**) the microstructure of the weld metal (W.M.), heat-affected zone (H.A.Z.), and base metal (B.M.) zones.

**Fig. 13:**
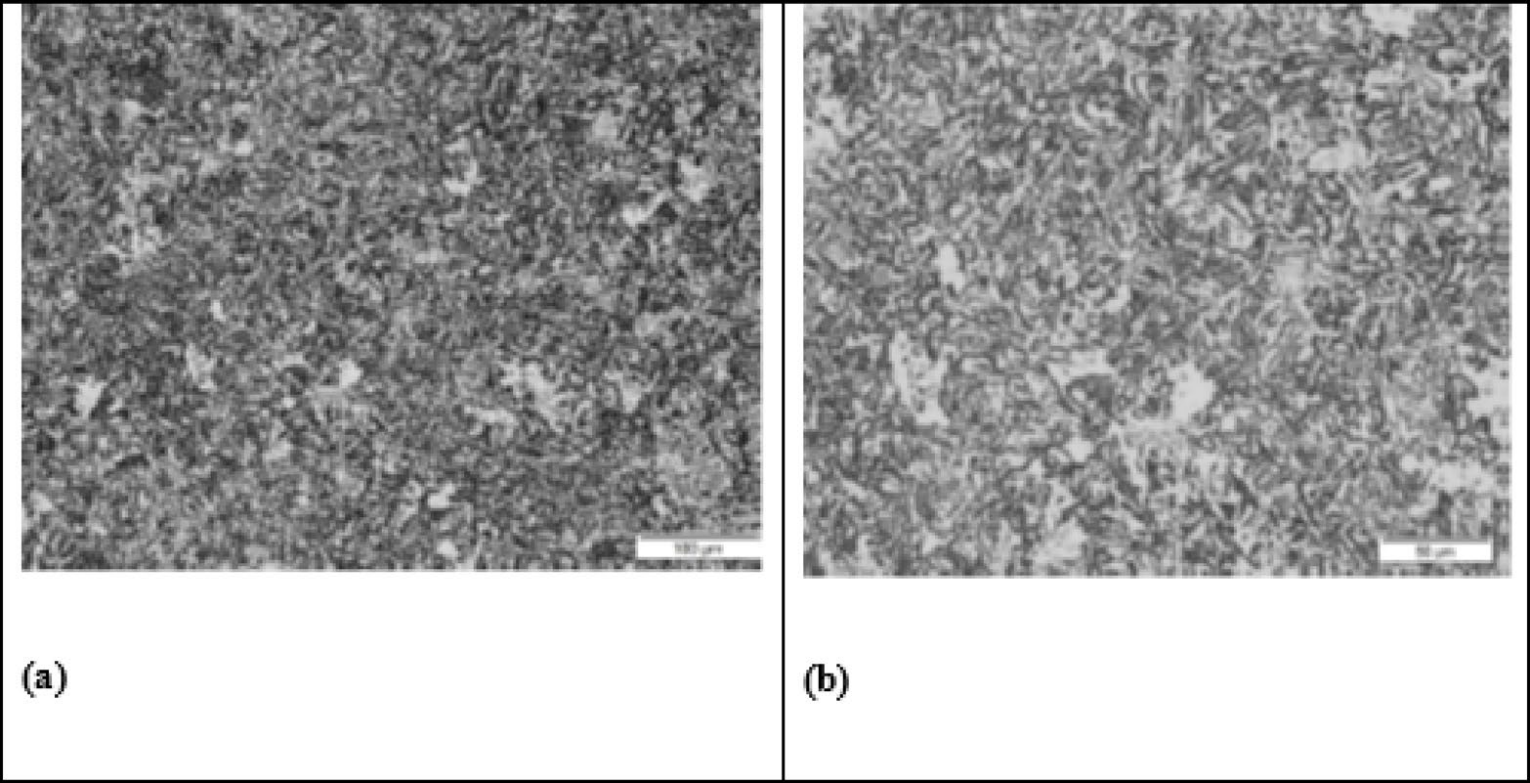
Weld metal microstructure of the joint welded by solid wire, (**a**) ER 70 S-3, at the mid-throat of the joint, and (**b**) at the center of the weld metal.

**Fig. 14:**
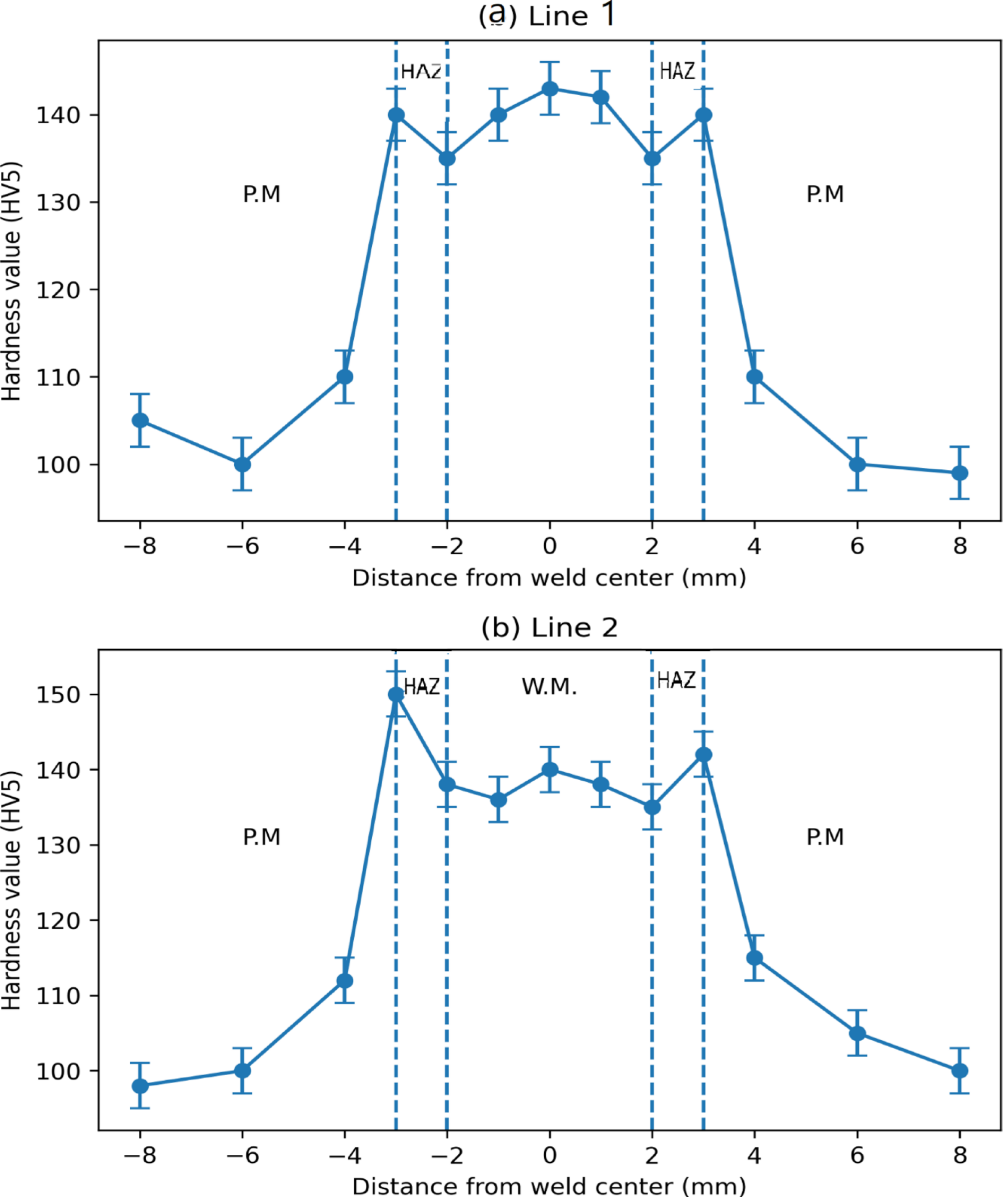
Hardness profile at weld metal (W.M.), heat-affected zone (H.A.Z.), and parent metal (P.M.) of GTPFAW Joint at (**a**) line 1 and (**b**) at line 2 for weld metal of GTPFAW Joint. Error bars represent the standard deviation of microhardness measurements.

A sufficient application of the powder filler, along with a satisfactory level of fusion and penetration of the weld metal utilizing the GTPFAW process, is illustrated in Fig. 9. Pre-welding visual assessments indicated a satisfactory distribution of the powder filler within the groove at the first pass, as demonstrated in Fig. 9(a). A sufficient degree of fusion bonding between the solidified weld pass and the base metal was observed, as corroborated in Fig. 9 (b and c). The penetration of the weld root was deemed acceptable, as evidenced by Fig. 9(d). Moreover, the height of the weld metal cap corresponding to the executed joint was considered acceptable, achieving an optimal toe blend. Furthermore, the application of the dye penetrant examination confirmed the nonexistence of surface porosity or superficial fractures such as surface cracks. The radiographic films interpretation indicated that the joint was devoid of internal defects, resulting in its acceptance. Table 6 delineates the compositional percentages of the weld metal located in the midsection, which substantiates a homogeneous blend and adequate distribution of elemental powder throughout the welding process. Moreover, a comparative analysis of the data presented in Table 6 for both specimens across the entire length of the welded GTPFAW joint reveals no compositional variations from one level to another within the joint length attributable to precipitation or segregation. All tensile specimens exhibited fractures outside the weld region in the parent metal. The tensile properties and fracture locations of all evaluated specimens are detailed in Table 7. Fracturing within the plate metal indicates that the resultant weld metal possesses a greater strength than the indicated fracture strength values of the parent metal, meaning the weld exhibits tensile overmatching. Figure 10 showcases one of the fractured specimens as a representative example. The macrographic examination result of the specimen, oriented perpendicular to the weld axis at the widest cap, is shown in Fig. 11. The macrostructure demonstrates commendable penetration and fusion of the side walls, thereby illustrating the homogeneity of the solidified weld metal along with acceptable inter-pass fusion. Additionally, the weld metal appears to be devoid of defects, alongside a narrow heat-affected zone (H.A.Z.). Microstructural morphologies across various zones along the weld metal and joint cross-section are shown in Fig. 12. A representation of the microstructure of the resultant weld metal of the GTPFAW joint at the root and cap zones is illustrated in Fig. 12(a and b), respectively. The microstructure observed at the mid-throat of the weld is evident in Fig. 12(c and d). The narrowing of the resultant H.A.Z., measuring only 0.8 mm, has been substantiated as indicated by the distinct grain boundaries revealed in Fig. 12(e). Thus, the supplementary benefit of the GTPFAW methodology lies in its ability to yield a restricted heat-affected zone (H.A.Z.). Furthermore, notwithstanding the solid wire’s composition of grain-refining constituents such as copper (Cu), zirconium (Zr), and titanium (Ti)^17–20^, the microstructural characteristics derived from the powder exhibit a significantly finer structure compared to those generated from the solid wire. This distinction is particularly observable when juxtaposing the resultant microstructure of the weld metal produced via the GTPFAW technique, as illustrated in Fig. 12(c and d), with that obtained from the solid wire utilizing the conventional GTAW/TIG process depicted in Fig. 13(a and b), respectively. Additionally, the grain size analysis conducted using the ImageJ software on the resultant GTPFAW weld metal indicates an average grain size of 6.8 μm (Standard Deviation = 9.5). Conversely, the image processing of the resultant microstructure from the solid wire weld metal verifies that the average grain size is 11 μm, which exceeds that of the powder-derived weld metal. Representative hardness profiles derived from average measurements are illustrated in Fig. 14(a and b), which elucidates that the weld metal exhibits a consistent hardness within the range of 135–143 HV5 across its entire cross-section.

**Table 6:** Chemical composition of the GTPFAW resultant weld metal in wt%.

	C	Si	Mn	S	*P*	Cr	Ni	Mo	Fe
Specimen 1	0.08	0.4	1.1	0.01	0.017	0.18	0.17	0.16	97.8
Specimen 2	0.08	0.4	1.1	0.006	0.005	0.2	0.1	0.15	97.9

**Table 7:** Tensile test results of the GTPFAW Joint.

Specimen no.	Tensile strength (MPa)			Fracture location	Note
	Fracture strength	Mean of fracture strength	S.D. of fracture strength		
1	518.6	468.66 (a)*	26.47 (b)*	Base metal	(c)*
2	470.5				(c)*
3	456.2				
4	455.3				
5	442.7				

(a)* Mean fracture strength of the welded joint, since the mean is related to the properties of the plate, as the fracture occurs in the parent metal itself, away from the resulting weld metal.

(b)* Standard deviation (SD) of fracture strength of the welded joint, since the SD is related to the properties of the plate, as the fracture occurs in the parent metal itself, away from the resulting weld metal.

(c)* Tensile strength is higher than the parent metal strength, as remarked in the plate certificate (459 MPa).

## Discussion

### Integrity of the welded structure

**Fig. 15:**
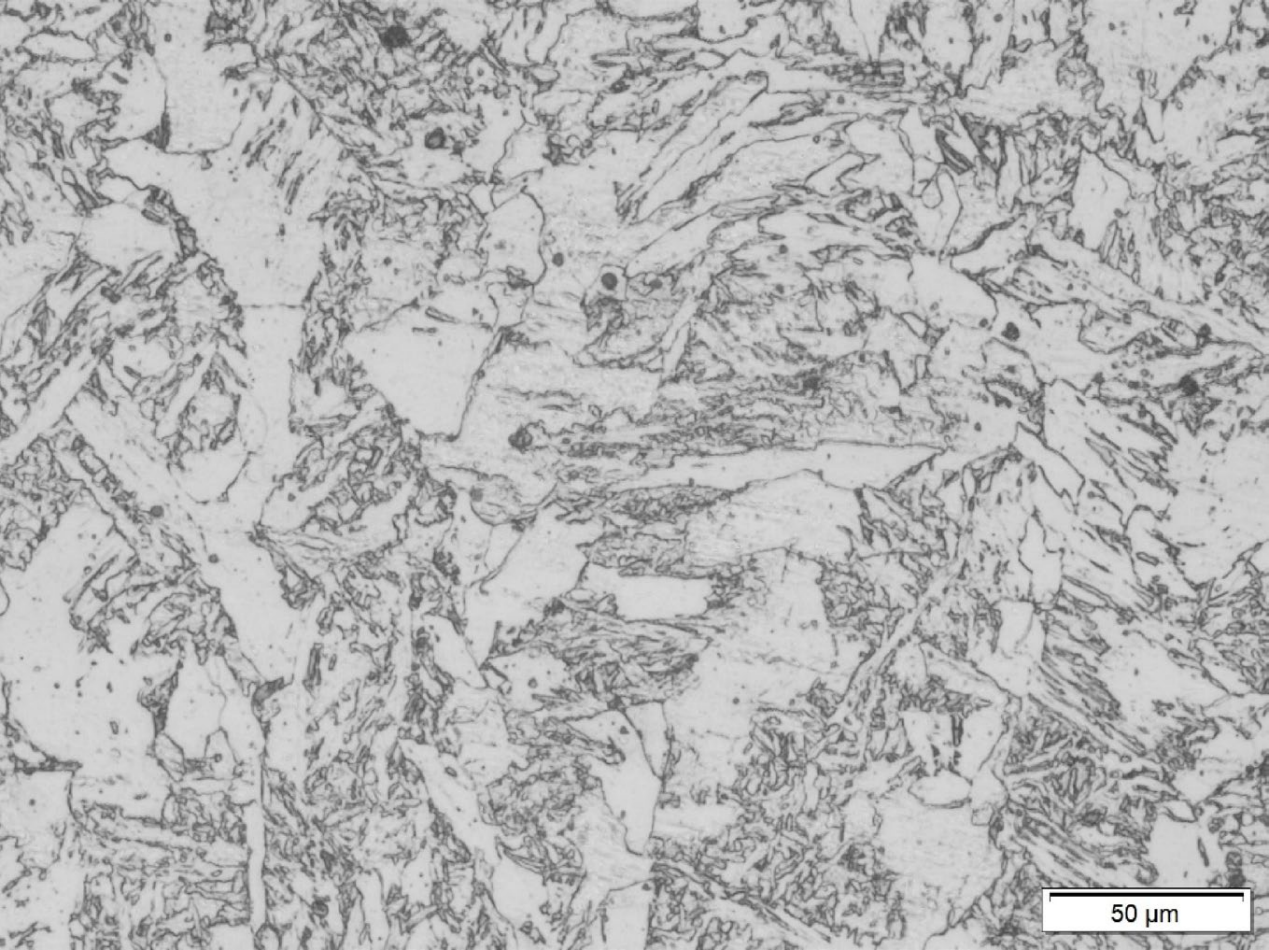
GTPFAW weld metal photomicrograph revealing the absence of microporosities.

**Fig. 16:**
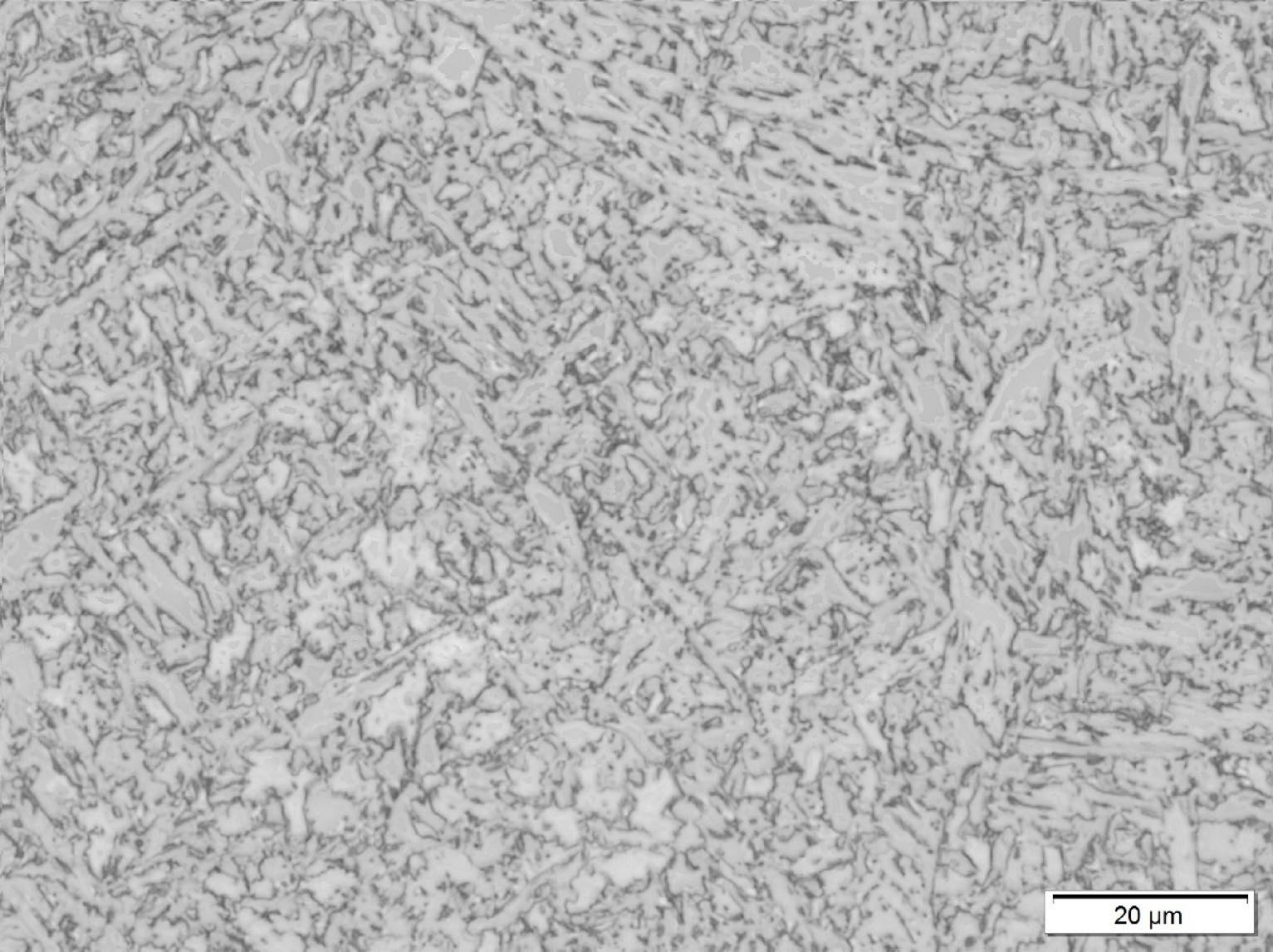
GTPFAW weld metal photomicrograph revealing the absence of voids and fusion defects.

The integrity of the welded structure throughout its operational lifespan is primarily contingent upon the degree of defect presence within its weld joints. The existence of weld defects compromises the designed mechanical strength requisite for the welded joints, potentially rendering the structure susceptible to failure under severe operational conditions. In general, the term weld defect is delineated as the occurrence of discontinuities that exceed allowable limits within the weld region. Based on the findings from non-destructive testing examinations, it is ascertainable that the visual assessments conducted during and after the welding operation substantiated the efficacy of the GTPFAW technique in powder melting for each welding pass, as well as the satisfactory fusion achieved between the solidified weld passes and the base metal, as illustrated in Fig. 9(b and c). An appropriate cap height and width, alongside adequate root penetration, can also be realized through this newly refined methodology, as evidenced in Fig. 9 (d) pertaining to joint penetration. Typically, the characteristics of a joint may be influenced by the weld configuration, for instance, the geometry of the weld toe, which impacts fatigue endurance, as articulated by B. Pollard and R. J. Cover^21^, who elucidated that an increased toe angle (and consequently a diminished reinforcement angle) correlates with reduced fatigue strength. In the present study, the GTPFAW technique yielded a weld cap characterized by a toe blend angle of less than 15° (with a reinforcement angle exceeding 165°), thereby indicating that the resultant joint can be deemed to demonstrate superior fatigue strength. Moreover, visual inspections have unveiled two additional significant advantages of the GTPFAW method that are highly advantageous in construction applications. The first advantage is the complete absence of spatter, while the second pertains to the minimization or elimination of the number of stop/start passes, thereby circumventing poor pass stop/start conditions, which are recognized as stress concentrators and consequently detrimental to joint efficiency. The outcomes of the dye penetrant testing corroborated the visual evaluations, particularly in confirming the absence of undercut imperfections. Furthermore, the results from radiographic testing have validated the integrity of the internal coherence of the solidified weld metal. Generally, the occurrence of cracks within weld joints can be attributed to significant disparities in stress concentrations, which may arise from inadequate fit-up, improper edge preparation, or insufficient welding protocols. The phenomenon of hot cracking or cold cracking in welded joints predominantly results from solidification/thermal behavior or the entrapment of hydrogen, respectively. Hydrogen can be absorbed from either the arc atmosphere or the filler material. The propensity for either of these cracking mechanisms was not visually detected or evidenced by dye penetrant and radiographic examinations, notwithstanding the employment of unbaked powder and the absence of heat treatment prior to or following the welding process. A shrinkage cavity may potentially develop within the weld metal zone as a consequence of the non-uniform retractility/shrinkage of the welding layers. Additionally, weld metal porosities and blowholes predominantly arise from the entrapment of gases within the weld during the solidification process. The radiographic examinations encompassing the entirety of the joint length have not revealed the presence of shrinkages, cavities, or porosities. This outcome from the radiographic imaging has substantiated and corroborated the feasibility of achieving a weld metal devoid of these defects when employing the novel welding technique. The radiographic findings confirmed the absence of detectable porosity or fusion defects in the resultant GTPFAW weld metal, which are corroborated by the microscopic analysis illustrated in Figs. 15 and 16, which verified the nonexistence of microscopic void defects within the weld metal. The presence of nitrides, oxides, and phosphorus compounds within the solidified flux trapped in the weld leads to the formation of slag inclusions. Such inclusions are detrimental to the mechanical properties of the weld, particularly its ductility. Notably, the joint radiographs did not indicate any such slag inclusions. A distinctive feature of the GTPFAW method was effectively illustrated through macrographic investigation, specifically the prevention of overlap defects. A gradual fusion with the base metal was achieved, characterized by an optimal toe blend. The smooth blending of weld toes is a critical factor in enhancing the resistance of welded joints against both fatigue and crevice corrosion. As evidenced by the macrostructure of the resultant GTPFAW joint, adequate fusion and the absence of all varieties of incomplete fusion have been confirmed. Both excessive penetration and insufficient penetration are viewed as undesirable in the majority of weld joints. Excessive penetration is recognized as a contributor to turbulent flow, which subsequently results in erosion, while insufficient penetration catalyzes stress concentration and cracking. In relation to the powder joint, sufficient penetration has been demonstrated through both visual and macroscopic assessments.

### Improved microstructure characterizations of the weld metal

**Fig. 17:**
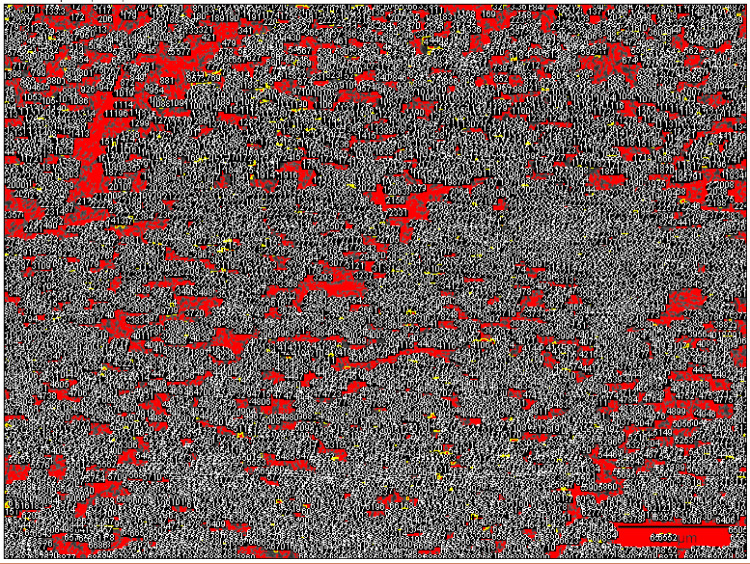
ImageJ-1.46r output for GTPFAW weld metal morphology analysis.

**Fig. 18:**
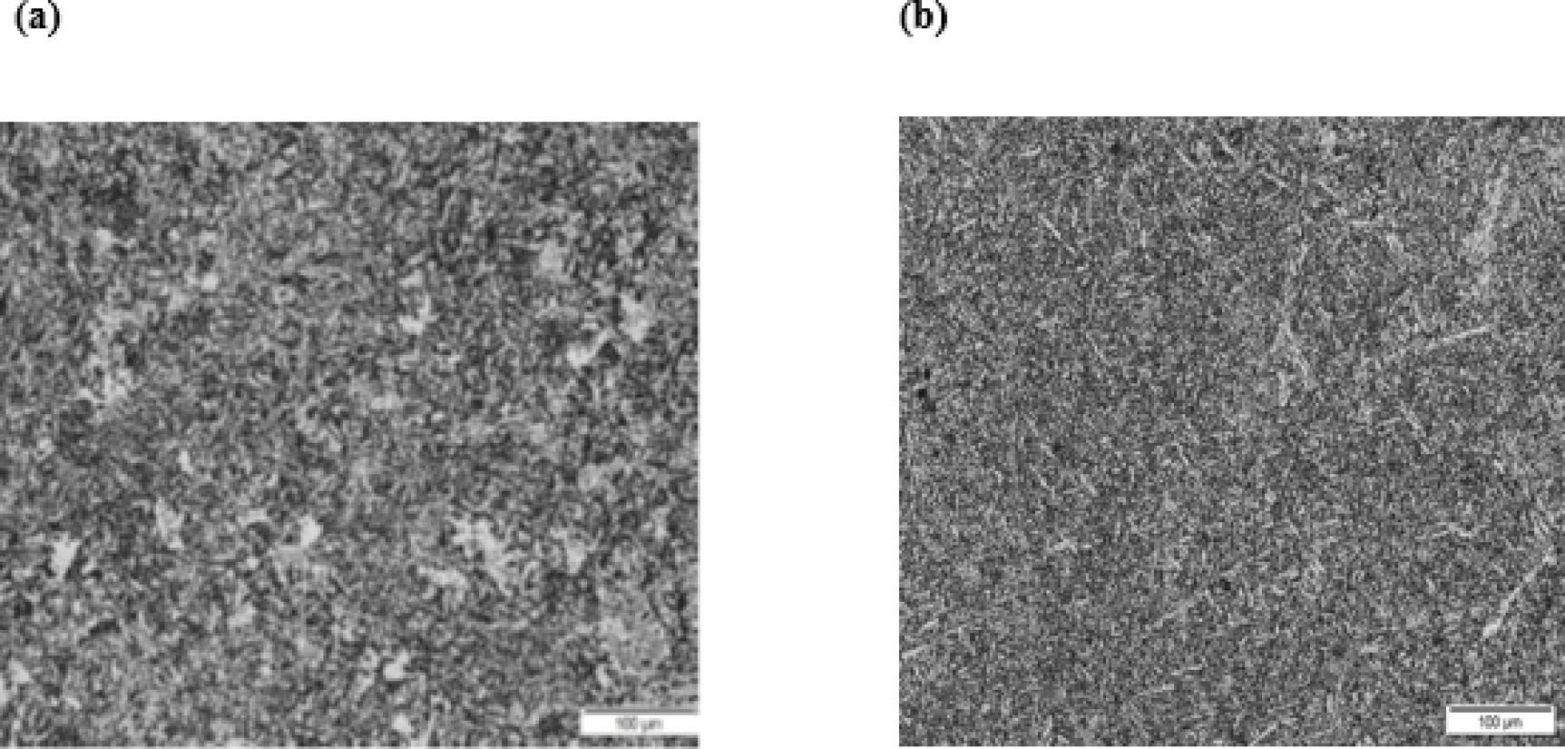
Various microstructural weld metals produced by solid wire and powder filler techniques, revealing the amount of acicular ferrite: (**a**) utilizing the conventional GTAW process and 2.4 mm ER 70 S-3 solid wire; and (**b**) utilizing the novel GTPFAW process using powder filler metal in micron size matching with ER 70 S-3 composition.

It has been revealed that the microstructure of the resultant GTPFAW weld metal exhibits a significant ferrite phase characterized by acicular morphology^22–27^. The resultant acicular ferrite is ultrafine and can be regarded as finer than that produced from the application of the micro-jet welding technique for joining carbon steel grade S 235 using a (GMAW) wire of 1.2 mm diameter, argon (Ar) gas for shielding, and various cooling gases, such as nitrogen and helium, which was articulated by D. Hadrys et al.^27^ as an advanced methodology for achieving an ultra-fine microstructure. It has been previously documented that the proportion of acicular ferrite morphology in ferritic weld metal produced via solid wire and traditional welding processes, including SMAW, SAW, MIG, and MAG, peaks at 55%. Specifically, the conventional GTAW/TIG welding process, which employs a solid wire filler metal, yields a maximum ferrite morphology content of 48%, attributed to the significantly lower concentration of oxygen in the deposited weld metal^28^. T. Węgrzyn et al.^26^ indicated that the primary objective for researchers is to elevate the acicular ferrite (AF) content in the resultant weld metal above 65%, as this has a beneficial impact on the mechanical properties^27^. It is imperative to emphasize that the resultant weld metal generated by the innovative GTPFAW welding technique exhibits a volume fraction of acicular ferrite exceeding 65% for carbon steel weldments. This quantification can be readily corroborated through the microstructural analysis outcomes of the GTPFAW weld metal morphology and the assessments conducted using ImageJ-1.46r software, as depicted in Fig. 17. In accordance with Fig. 18, which elucidates a comparative analysis of various microstructural configurations and the corresponding proportions of acicular ferrite present in carbon steel; S275 JR joints produced in this investigation utilizing solid wire and powder filler methodologies, designated as conventional GTAW and the innovative GTPFAW respectively, it can be underscored that although the microstructural examination was executed using optical microscopy, as opposed to scanning electron microscopy (SEM), which affords enhanced resolution and increased depth of field for meticulous scrutiny of grain boundary morphology, or electron backscatter diffraction (EBSD) which generates crystallographic orientation maps for precise quantification of grain size and misorientation; the superior microstructure and the maximal concentration of acicular ferrite have been substantiated as a consequence of employing the proposed novel GTPFAW methodology, and it is anticipated that this outcome may be further refined and optimized if investigations are conducted utilizing either SEM or EBSD techniques. A parallel conclusion can be drawn when contrasting the results derived from the application of the GMAW micro-jet cooling method^27^. Consequently, the weld metal produced through GTPFAW, characterized by a substantial presence of acicular ferrite, correspondingly signifies an enhancement in confidence regarding superior impact toughness properties^27,29^. Regarding the refinement of weld microstructure facilitated by the new method, this phenomenon can be attributed to the incorporation of a micro powder mixture as the filler metal. Greer^30,31^ posited that there exists an inverse relationship between the number of elements present in an alloy and the probability of the alloy achieving a viable crystal structure during solidification. In a similar vein, when multiple elements are incorporated for a phase transformation, the kinetics of nucleation or growth become sluggish, as the genesis of a new phase necessitates the cooperative diffusion of elements to achieve the requisite composition, and the efficiency of cooperative diffusion diminishes with an increasing number of elements involved. Building upon this principle, one may examine the kinetic distinctions between the micro powder filler and the solid wire filler. From the volumetric analysis contrasting the solid wire of millimeter dimensions (1.2 mm in diameter) with the micron-sized particles of the powder filler, it becomes evident that the alloy wire is predisposed to attain a more homogeneous melting compared to the elemental powder, given that an elemental atom from its liquid phase necessitates a protracted duration to diffuse cooperatively to create a homogeneous alloy melt. As both the melting and rapid solidification processes during arc welding occur expeditiously, while the elemental powder requires a longer duration to achieve a homogeneous melt, the transition of this homogeneous melt into the ferrite phase will inevitably be delayed, occurring at lower temperatures than those observed with alloy wire. Consequently, the micro powder amalgamation/mixture exhibits a superior nucleation density and a reduced grain size in comparison to the alloyed solid filler wire. This principle and underlying mechanism may also be extended to other rapid melting and solidification phenomena originating from elemental powders. This assertion is further substantiated by the observations that (1) while the thermal input values during the welding process for both solid wire and powder filler joints are relatively comparable as indicated in Tables 3 and 5, the resultant weld metal derived from the powder exhibits a significantly finer microstructure than that produced by solid wire. (2) Even though the number of welding passes in powder welding exceeds that in solid wire, thereby indicating that the weld metal is subjected to greater thermal exposure in the powder process, the grain size of the weld metal fabricated using the powder remains the most refined. (3) Although the cross-sectional area designated for thermal dissipation in the transverse and lateral directions remains invariant due to the constancy of joint geometry parameters, such as root face, root opening, bevel angle, and groove angle across both experimental conditions, the resultant powder weld exhibits a significantly reduced size in comparison to the solid wire weld. (4) Notwithstanding the backing type employed in the powder welding experiment, which contributed to heat retention and diminished thermal dissipation-thereby averting rapid cooling due to the substantial thermal mass of the backing material-the grain size resulting from the powder welding process was nonetheless inferior to that observed in solid wire welding.

### Improved mechanical properties of the welded joint

In order to validate a welding procedure and assess the quality and characteristics of the weld metal, a transverse weld tensile test was employed to verify that the strength of the weld metal is at least equivalent to that of the parent metal. The significance of the transverse weld tensile test lies in its ability to evaluate the properties across the entire weld region that is oriented perpendicularly to the weld seam, encompassing the weld, heat-affected zone (H.A.Z.), and the unaffected base metals. This heterogeneous region is subject to greater influence from the variables associated with the welding procedure. Consequently, tensile strength can be regarded as the predominant mechanical property for evaluating the weldability and overall quality of the joint produced through any welding method. Given its importance, the transverse weld tensile test has been regarded as the primary criterion for assessing the efficacy of the presented GTPFAW technique in achieving successful welding and appropriate joint integrity, based on the location of fracture and the ultimate tensile strength of the resultant joint. Through the tensile testing conducted, all tensile outcomes unequivocally substantiated the robust adhesion between the powder filler metal and the parent metal, which culminated in enhanced weld joint integrity and fracture occurrence within the base metal. Thus, the tensile test proved the success and validity of welding using the modified GTPFAW method and confirmed the efficiency of this novel method in getting a welded joint with high mechanical strength. Consequently, the tensile test validated the efficacy and reliability of the welding technique employing the modified GTPFAW methodology, thereby affirming the proficiency of this innovative approach in achieving a welded joint characterized by superior mechanical strength. The findings derived from the tensile test instilled confidence that the resultant joint attributes engendered by the GTPFAW process are in substantial concordance with the stringent requirements delineated by international welding qualification standards and codes (such as AWS D1.1, ASME IX, and/or BS EN ISO 15614-1, which stipulate that the tensile strength of the test specimen shall not fall below the designated minimum value for the parent metal). Hence, it necessitates that fracture occurrence transpires in a region extraneous to the weld zone on standardized tensile specimens whose gauge encapsulates all areas of the weld. The hardness of the parent metal was ascertained to be within the range of 98–115 HV5. In contrast, the hardness metrics for the weld metal exhibited remarkable uniformity across its various zones when traversing from root to cap and between bevels, where they were recorded in the range of 135–143 HV5.

The hardness measurements of both the parent and weld metals can be interpreted as being contingent upon their involvement in the ferritic phase of each. It can be elucidated that the elevation in hardness values of the weld relative to the base metal is attributable to the predominance of a fine acicular ferrite microstructure within the weld metal. The hardness values observed in the heat-affected zone surpass those recorded in the base metal (138–150 HV5 as opposed to 98–115 HV5), a phenomenon that can be rationalized by taking into account the rapid cooling experienced in the HAZ, particularly in regions where the joint lacked preheating.

### Potential industrial environment advantages of the GTPFAW method

As global interest in environmental sustainability intensifies, the field of welding is increasingly included within this focus. In the context of environmentally friendly welding practices, it is imperative that the welding techniques chosen are evaluated in order to mitigate the carbon footprint. Regrettably, a significant proportion of traditional electric arc welding techniques employed for metal joining, such as SMAW, SAW, and Flux-Cored Arc Welding (FCAW), release undesirable and detrimental gases, including carbon monoxide and carbon dioxide^32–36^. Conversely, the newly introduced GTPFAW method does not generate these emissions, as it does not utilize flux. The operation of melting furnaces, such as Electric Arc Furnaces (EAF), necessitates electrical energy consumption for the production of solid wire from alloyed raw metals, as well as for wire formation and sizing through processes such as rolling and drawing. Given that the electrical energy is predominantly derived from the combustion of fossil fuels, its extensive utilization poses significant environmental repercussions^36–39^. Consequently, employing granulated filler metal as opposed to solid forms, as is common in traditional arc welding techniques, results in a reduction in the energy required for the fabrication of solid welding wires. Thus, it can be posited that the introduced welding method contributes to the realization of the climate objectives delineated in the 2030 Agenda for Sustainable Development^40–42^.

## Conclusions

The weldability of the innovative Gas Tungsten Powder Filler Metal Arc Welding (GTPFAW) process was rigorously investigated and appraised for its applicability in structural welds on S 275 JR steel, employing a filler metal composed of a micro-powder mixture that aligns with the chemical composition of ER 70-S3 filler metal. Additionally, solid wire ER 70-S3 was utilized in conjunction with a traditional GTAW/TIG technique to facilitate a comparative assessment of the resulting microstructural morphology relative to that of the aforementioned process. The conclusions are articulated as follows:

- The GTPFAW method has evidenced its technical feasibility for proficient joining.
- GTPFAW resultant joint demonstrated acceptable structural integrity without evidence of critical discontinuities. The quality of the weld was substantiated through non-destructive evaluation (NDE), which included visual inspection, dye penetrant, and radiographic methods.
- The root penetration was deemed satisfactory. (The structural integrity of the welded joint is fundamentally reliant upon achieving complete root penetration, as inadequate fusion at the root serves as a stress concentrator and precipitates premature failure).
- Fracture analysis indicated a predominant failure occurring at the parent metal, suggesting that the bonding strength surpasses that of the base metal.
- The transverse tensile characteristics of the resulting joint exhibited adherence to the stipulations set forth by international welding qualification standards and codes (e.g., AWS D1.1 and/or ASME IX, as it is mandated that the fracture should transpire within the base metal, rather than within the weld metal.
- A homogeneous melting of the powder filler metal was confirmed, resulting in weld metal characterized by ultrafine grain sizes that are significantly finer than those produced using conventional solid filler.
- GTPFAW exhibited a substantial presence of acicular ferrite, which is recognized as.

the most advantageous phase in the weld metal of carbon and low-alloy steels.

- The uniform hardness distribution observed in the weld metal was congruent with a fine and consistent microstructure.
- Empirical investigations facilitated the enhancement and corroboration of the reconditioning technology for the metal composition utilized in filling applications. The GTPFAW process has exhibited adaptability in the formulation and implementation of appropriate compositions tailored to welding specifications, which can also be regarded as an ecologically sustainable welding technique, aligning with the stipulations of the 2030 Agenda, in conjunction with its economic advantages in the generation of specific chemical formulations that are not readily available on-site. The method developed has demonstrated reliability within the production milieu, thereby ensuring a high standard of output quality.

## Supplementary Information

Below is the link to the electronic supplementary material.

Supplementary Material 1

## Data Availability

All data generated or analyzed during this study are included in this published article.
