# Supplementary material for: Novel gas tungsten powder filler metal arc welding process
Source: Sci Rep. 2026 Aug 5;16:24166. doi: 10.1038/s41598-026-64307-z (PMC13443633; doi:10.1038/s41598-026-64307-z)
Supplement: Supplementary file 1 [file 41598_2026_64307_MOESM1_ESM.docx]

Empirical methodologies are currently being implemented to mechanize the novel GTPFAW technique, which has received patent recognition from Taiwan MOEA (R.O.C.) – Innovation No. I677398) and from the Arab Republic of Egypt (MOHESR & ASRT – Innovation No. 30333). The mechanization encompasses the powder delivery and feeding system, as well as torch manipulation, which is governed by mechanical appendages, the movements of which are meticulously regulated by a processing unit and software specifically tailored for this application, as illustrated in the following sequence.

|  |  |
| --- | --- |
|  |  |
|  | |

Empirical methodology to mechanize the novel GTPFAW technique.
